# The Association between *Mycoplasma pneumoniae* Genotype and Cutaneous Disease

**DOI:** 10.3390/microorganisms11010205

**Published:** 2023-01-13

**Authors:** Jasna Rodman Berlot, Tatjana Mrvič, Mitja Košnik, Darja Keše

**Affiliations:** 1 Department of Paediatric Pulmonology, University Children’s Hospital, University Medical Centre Ljubljana, 1000 Ljubljana, Slovenia; 2 Department of Infectious Diseases, University Medical Centre Ljubljana, 1000 Ljubljana, Slovenia; 3 University Clinic of Respiratory and Allergic Diseases Golnik, 4204 Golnik, Slovenia; 4 Faculty of Medicine, University of Ljubljana, 1000 Ljubljana, Slovenia; 5 Institute of Microbiology and Immunology, Faculty of Medicine, University of Ljubljana, 1000 Ljubljana, Slovenia

**Keywords:** *Mycoplasma pneumoniae*, genotype, cutaneous disease, children

## Abstract

*Mycoplasma pneumoniae* (*Mp*) can cause several extrapulmonary manifestations, most frequently dermatological ones. It is largely unknown whether *Mp* genotype determines *Mp*-induced cutaneous disease. The aim of our study was to assess the association between *Mp* genotype and this clinical outcome. We performed a retrospective study of children referred with signs of acute *Mp* infection from 1 January 2014 to 31 December 2014. We compared the characteristics of children presenting as cutaneous disease, upper (URTI) and lower respiratory tract infection (LRTI). In addition, we separately analyzed the data of patients presenting with *Mp*-induced cutaneous disease. We evaluated data from 435 patients (mean age 7.3 years, SD 3.4 years; 52.0% boys) who had *Mp* PCR-positive pharyngeal swab, P1 genotype and/or multilocus variable-number tandem-repeat analysis (MLVA) genotype defined and no viral co-detection, presenting as cutaneous disease (38/435), URTI (46/435) or LRTI (351/435). The majority of patients had urticarial (55%, 21/38) or maculopapular eruptions (37%, 14/38). We found no association between *Mp* genotype and clinical outcome of cutaneous disease, nor any specific dermatological presentation. In the group with cutaneous disease, 18% (7/38) required hospital admission because of rash. We found that infection with MLVA-3,6,6,2 strains was more common in admitted patients than in outpatients (40% vs. 4%, *p* = 0.017) and significantly affected the likelihood of hospital admission in a logistic regression model. The results of our cohort study suggest that *Mp* genotype does not determine *Mp*-induced cutaneous disease or a specific dermatological presentation. Nevertheless, infections with certain MLVA strains could induce more severe cutaneous disease requiring hospitalization.

## 1. Introduction

*Mycoplasma pneumoniae* (*Mp*) is a common cause of respiratory tract infections (RTI) in children and adults worldwide [1,2,3]. Nevertheless, it can also cause several extrapulmonary manifestations involving all the major organ systems [1,4]. The most prevalent form of extrapulmonary manifestations are the dermatological ones. It is estimated that up to one-third of patients with *Mp* infection have some cutaneous form of disease [5,6,7,8,9,10].

*Mp* strains can be classified into different genotypes by using several typing methods, most frequently P1 typing, multilocus variable-number tandem-repeat analysis (MLVA) and multilocus sequence typing (MLST) [11,12]. P1 typing separates the isolates into two major subtypes, P1 type 1 and P1 type 2, according to nucleotide differences in two repetitive elements (RepMP2/3 and RepMP4) in the MPN141 gene that codes for the P1 adhesion protein [13,14,15]. It was the most frequently used genotyping method until newer methods were developed. In comparison to P1 typing, MLVA and MLST offer a more discriminative categorization of isolates. MLVA typing separates the isolates based on the variable copy numbers of tandem repeat sequence at specific loci [11,16], while MLST offers an even higher discriminative power with the aid of whole-genome sequencing analysis [17].

We recently described an association between P1 and MLVA genotype and severity of lower respiratory tract infections (LRTI) in children [18,19]. However, it is largely unknown whether a specific *Mp* genotype is associated with a specific clinical outcome, such as cutaneous disease. Moreover, it is unknown whether a specific *Mp* genotype is associated with specific dermatological presentation.

The aim of this study is to present our experience with dermatological manifestations of *Mp* infection during a recent *Mp* epidemic in 2014 [20], with special attention to the association between *Mp* genotype and *Mp*-induced cutaneous disease. The hypothesis of our study is that a specific *Mp* genotype is associated with clinical outcome, specific dermatological presentation and severity of *Mp*-induced cutaneous disease.

## 2. Materials and Methods

### 2.1. Study Subjects

Children younger than 18 years, referred to University Children’s Hospital and Department of Infectious Diseases Ljubljana, Slovenia, with clinical signs of acute *Mp* infection from 1 January 2014 to 31 December 2014, were tested for *Mp*.

All patients who were PCR positive for *Mp* in pharyngeal swabs and in whom the P1 and/or MLVA genotype was successfully defined were identified from a laboratory database and included in the study. We excluded cases with a viral co-detection.

The National Medical Ethics Committee of the Republic of Slovenia approved the protocol for this study (No 0120-8/2018/4 and No 0120-244/2021/3).

### 2.2. Study Design

We performed an observational retrospective study to determine whether *Mp* genotype is associated with *Mp*-induced cutaneous disease in children.

The data on age, gender, disease presentation, dermatological presentation, *Mp* genotype, macrolide susceptibility, interval between onset of disease and initiation of antibiotic therapy, laboratory biomarkers of inflammation, hospital admission, duration of hospital stay and data related to complications and treatment were collected for all patients.

The patients were further divided into groups according to the disease presentation; patients with cutaneous disease, patients with upper (URTI) or lower respiratory tract infection (LRTI), and their epidemiological characteristics and *Mp* genotype distribution were compared. Patients with other rare extrapulmonary manifestations were excluded from the study. Cutaneous disease was defined as an eruptive lesion, which involved skin. We present *Mp*-induced dermatological presentations reported previously in the literature [5,6]. LRTI diagnosis was made based on physical examination revealing pathological lung auscultation and radiographic appearance consistent with a diagnosis of LRTI, if an X-ray was performed. Patients were diagnosed with URTI if they had respiratory symptoms and did not fulfill LRTI criteria.

In addition, the group of patients with cutaneous disease was divided according to a specific dermatological presentation, and the characteristics of each group were evaluated and compared.

To better assess the possible factors influencing the severity of the disease, we compared the characteristics of patients hospitalized because of rash with those of outpatients.

### 2.3. Methods

Pharyngeal swabs were subjected to DNA isolation using MagNA Pure Compact (Roche Diagnostics, Mannheim, Germany) and later tested by *Mp* real-time PCR (ArgeneBioMerieux diagnostics, Marcy l’Etoile, France). The remainder of each PCR-positive sample was cultivated as described previously [20]. MLVA subtyping was performed by amplification of four variable-number tandem-repeat (VNTR) loci (Mpn13, Mpn14, Mpn15 and Mpn16) according to a standardized MLVA protocol [16]. P1 subtyping was performed using pyrosequencing, which targets the *Mp* MPN141 and MPN528a genes, while macrolide resistance was recognized by pyrosequencing two parts of domain V in the 23S rRNA gene [20].

Multiplex PCR was performed on the nasopharyngeal aspirate specimens to assess viral co-detection, including respiratory syncytial virus, influenza virus, parainfluenza virus, human bocavirus, adenovirus, metapneumovirus, rhinovirus, enterovirus and coronavirus.

### 2.4. Analysis

Continuous variables were presented as mean (SD) or median (IQR), where appropriate. Categorical variables were described with counts and percentages.

Continuous variables in two independent groups were compared using the independent samples T-test or the Mann–Whitney U-test, where appropriate, whereas continuous variables in multiple independent groups were compared using the one-way ANOVA or Kruskal–Wallis test, where appropriate [21]. Categorical variables were compared by using the Pearson chi-square test. We performed a multivariable logistic regression analysis to assess whether *Mp* genotype is associated with hospital admission because of cutaneous disease [22]. The differences were considered statistically significant when the *p* value was <0.05. Statistical computing was conducted in IBM SPSS Statistics (Version 28.0).

## 3. Results

**Figure 1:**
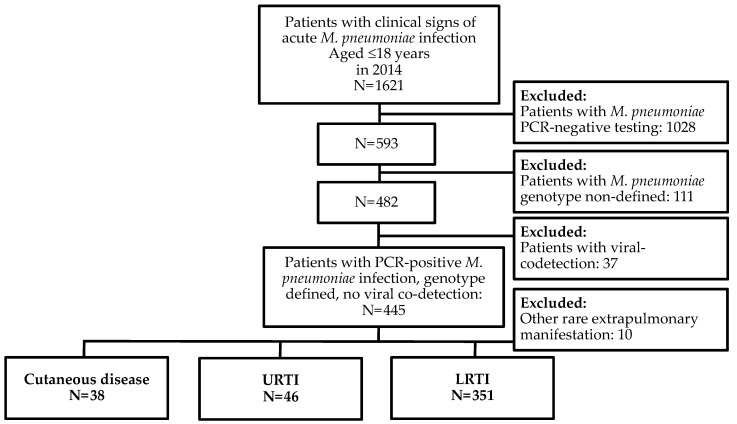
Study flowchart. Abbreviations: LRTI, Lower respiratory tract infection; MLVA, Multilocus variable-number tandem-repeat analysis; URTI, Upper respiratory tract infection.

During the study period, 1621 children were referred to our hospitals with signs of acute *Mp* infection. After applying the inclusion and exclusion study criteria, we evaluated data from 435 patients (mean age 7.3 yrs., SD 3.4 yrs.; 52.0% boys) with PCR-positive acute *Mp* infection, P1 and/or MLVA genotype defined and no viral co-detection (Figure 1), presenting as cutaneous disease (38/435), URTI (46/435) or LRTI (351/435).

The two main P1 genotypes, P1 type 1 and P1 type 2, accounted for 74 and 26% of total isolates, respectively. MLVA typing revealed seven distinct MLVA types: MLVA-3,5,6,2 (10.6%, 34/322), MLVA-3,5,6,3 (0.3%, 1/322), MLVA-3,6,6,2 (12.7%, 41/322), MLVA-4,5,6,2 (0.3%, 1/322), MLVA-4,5,7,2 (72.4%, 233/322), MLVA-4,5,7,3 (3.4%, 11/322) and MLVA-5,5,7,2 (0.3%, 1/322). The majority of isolates (99.3%, 432/435) were macrolide susceptible, and three (0.7%) were found to be macrolide resistant (MR*Mp*). Of the MR*Mp*, infection with one MR*Mp* strain presented as cutaneous disease (3%, 1/38).

We compared P1 and MLVA *Mp* genotype distribution in children with cutaneous disease, URTI and LRTI (Table 1). We found no association between a specific *Mp* genotype and a specific disease presentation (Table 1). However, infections with only three MLVA strains presented with cutaneous disease, including MLVA-3,6,6,2, MLVA-4,5,7,2 and MLVA-4,5,7,3.

The two most common skin manifestations during the study period were urticarial (55%, 21/38) and maculopapular eruptions (37%, 14/38), accounting for 92% of all cutaneous disease (Table 2).

When comparing the two groups, we found that patients with urticarial rash were more often treated with antihistamines and were hospitalized more often because of skin disease (Table 3). We found no association between a specific *Mp* genotype and a specific dermatological presentation. Nevertheless, only infections with MLVA-4,5,7,2 strains occurred as maculopapular eruptions, while infections with all three MLVA strains could present as urticarial rash.

The characteristics of patients with rare skin manifestations (8%, 3/38) are listed in Table 4. No mucocutaneous eruptions, such as toxic epidermal necrolysis or Stevens–Johnson syndrome associated with *Mp* infection, were observed in our patients.

In the group with *Mp*-induced cutaneous disease, 18% (7/38) required hospital admission because of rash. When comparing the characteristics of patients admitted to the hospital to outpatients’ ones, we found an important difference in their age, treatment and distribution of MLVA-3,6,6,2 genotype in both groups (Table 5). Children who were hospitalized were younger and more frequently treated with antihistamines and systemic steroids. In addition, infection with MLVA-3,6,6,2 strains was more common in admitted patients.

The observed difference in hospital admission remained significant even after adjusting for age in a logistic regression model for the MLVA-3,6,6,2 genotype (Table 6).

## 4. Discussion

Cutaneous disease is the most common extrapulmonary presentation of *Mp* infection [1,5,6,7,8,9,10]. The factors determining disease presentation and severity of *Mp*-induced cutaneous disease are only partly understood, with undiscovered roles for both pathogen- and host-related factors. The aim of our study was to determine whether *Mp* genotype is associated with this specific clinical outcome in children. In the study period, 8.5% of all patients presented with *Mp*-induced cutaneous disease. The two most common dermatological presentations were maculopapular and urticarial eruptions, accounting for 92% of all cutaneous disease. Patients requiring hospitalization because of rash were those with rare *Mp*-induced cutaneous disease, such as Henoch–Schönlein purpura and erythema multiforme, as well as those with urticarial rash, mimicking allergic reaction, and were therefore frequently treated with antihistamines and steroids.

When comparing *Mp* P1 and MLVA genotype distribution in patients with cutaneous disease, URTI and LRTI, we found no association between a specific P1 and MLVA genotype and *Mp*-induced cutaneous disease. Moreover, we found no association between *Mp* genotype and a specific dermatological presentation. Although seven different MLVA types co-circulated during the study period, infections with only three MLVA strains presented with cutaneous disease, including MLVA-3,6,6,2, MLVA-4,5,7,2 and MLVA-4,5,7,3. Moreover, when assessing the factors associated with severe cutaneous disease requiring hospital admission, we found that infection with MLVA-3,6,6,2 strains was more common in admitted patients. In addition, after adjusting for age, MLVA-3,6,6,2 genotype significantly affected the likelihood of hospital admission because of cutaneous disease in a logistic regression model.

Several possible mechanisms have been proposed to play a role in *Mp*-induced cutaneous disease [4,5,23]. It is suggested that an indirect mechanism, in which the bacterium is not present at the site of inflammation, but immune modulation, such as autoimmunity or formation of immune complexes, is thought to play an important role [4,24,25].

Microbiological studies have shown that *Mp* genotypes may have different characteristics, which could potentially impact their virulence and inflammation [26,27,28]. Results from clinical studies have also shown that P1 and MLVA genotypes could influence the severity of LRTI [18,19,29,30]. However, limited clinical studies to date have investigated the influence of the *Mp* genotype on *Mp*-induced cutaneous disease. To our knowledge, our sample size is the largest cohort of patients with *Mp*-induced cutaneous disease addressing this question either in children or adults. In addition, to better assess the impact on disease severity of different genotypes, we included outpatients as well as hospitalized patients in our study. Similar to our study, results from recent research found no association between *Mp* genotype and clinical outcomes, such as cutaneous disease [31]. In comparison to their study, our cohort of patients is larger, also including patients younger than 3 years of age, with no viral co-detection, to better assess the role of *Mp* in disease presentation. Macrolide-resistant *Mp* has been reported to be more frequently associated with mucocutaneous disease, possibly as a result of stronger and more persistent inflammatory stimulation by MR*Mp* because of treatment failure [32]. In our study, we detected only a single MR*Mp* strain in a patient with cutaneous disease.

Our study has several limitations. First, even though the sample of patients is larger compared to that in previous studies, a relatively small number of patients presented with extrapulmonary manifestations, such as *Mp*-induced cutaneous disease and specific dermatological manifestations. In addition, a relatively small number of patients were infected with specific MLVA genotypes. Second, all of our patients were recruited from tertiary centers, which may have resulted in a disproportionate number of cases with more severe *Mp* infection. Nevertheless, no mucocutaneous eruptions, such as toxic epidermal necrolysis or Stevens–Johnson syndrome associated with *Mp* infection, were observed in our patients. Third, the retrospective design of our study limited data collection to the most commonly used clinical variables. However, our study focused mainly on the association between *Mp* genotype and cutaneous disease, which was not influenced by the study design. Although it may be difficult to draw any definite conclusions, this study provides valuable information on the association between *Mp* genotype and cutaneous disease. Future studies should investigate other inflammatory parameters, disease severity and host-related factors to better understand the pathogenic role of *Mp* genotype in this clinical outcome.

## 5. Conclusions

The results of our cohort study suggest that no specific *Mp* genotype predisposes children to *Mp*-induced cutaneous disease and a specific dermatological presentation. Interestingly, infections with few MLVA strains were associated with this clinical outcome. In addition, the results suggest that infection with certain MLVA strains could induce more severe cutaneous disease requiring hospital admission. Future studies are needed to better understand the role of *Mp* genotype in *Mp*-induced cutaneous disease.

## Figures and Tables

**Table 1:** Epidemiologic characteristics and *Mycoplasma pneumoniae* genotype distribution in patients with cutaneous disease, upper and lower respiratory tract infection. Data are presented as median (IQR) or as percentage (proportion of subjects).

	Cutaneous Disease	URTI	LRTI	Test Statistic, *p*
Subjects N	38	46	351	
Boys/girls (%)	50%/50%	63%/37%	51%/49%	*χ^2^*(2) = 2.54, 0.281
Age (years)	6.6 (IQR 4.6–9.8)	5.9 (IQR 4.1–9.4)	7.0 (IQR 4.9–9.9)	H(2) = 1.34, 0.511
<5 years 5–18 years	32% (12/38) 68% (26/38)	33% (15/46) 67% (31/46)	26% (93/351) 74% (258/351)	*χ^2^*(2) = 1.09, 0.579
*Mp* Genotype P1-1/P1-2 (%) MLVA type	82%/18%	70%/30%	74%/26%	*χ^2^*(2) = 1.63, 0.442
MLVA-3,6,6,2 MLVA-4,5,7,2 MLVA-4,5,7,3	10% (3/29) 86% (25/29) 4% (1/29)	18% (7/38) 66% (25/38) 3% (1/38)	12% (31/255) 72% (183/255) 4% (9/255)	*χ^2^*(2) = 1.33, 0.514 *χ^2^*(2) = 3.65, 0.162 *χ^2^*(2) = 0.08, 0.960

Abbreviations: IQR, Interquartile range; LRTI, Lower respiratory tract infection; MLVA, Multilocus variable-number tandem-repeat analysis; URTI, Upper respiratory tract infection. Continuous variables were compared using the Kruskal–Wallis test, whereas categorical variables were compared using the Pearson chi-square test.

**Table 2:** Frequency of dermatological presentations of *Mycoplasma-pneumoniae*-induced cutaneous disease.

Dermatological Presentation	Frequency
Urticarial eruptions	55.3% (21/38)
Maculopapular eruptions	36.8% (14/38)
Erythema multiforme	2.6% (1/38)
Henoch–Schönlein purpura	2.6% (1/38)
Pityriasis rosea	2.6% (1/38)

**Table 3:** Characteristics of patients with maculopapular and urticarial *M. pneumoniae*-induced cutaneous disease. Data are presented as median (IQR) or as percentage (proportion of subjects). Significant differences (*p* < 0.05) are highlighted in bold.

	Maculopapular Eruptions	Urticarial Eruptions	Test Statistic, *p*
Subjects N	14	21	
Boys/girls (%)	43%/57%	48%/52%	*χ^2^*(1) = 0.08, 0.782
Age (years)	6.5 (IQR 4.6–10.0)	6.5 (IQR 4.4–8.1)	U(1) = 129.0, 0.544
<5 years 5–18 years	29% (4/14) 71% (10/14)	38% (8/21) 62% (13/21)	*χ^2^*(1) = 0.34, 0.561
CRP (mg/L)	14.0 (IQR 4.0–32.3)	19.5 (IQR 4.5–47.0)	U(1) = 92.5, 0.413
WBC (×10^9^/L)	9.1 (IQR 6.4–12.3)	10.0 (IQR 7.5–14.2)	U(1) = 96.5, 0.519
Hospitalization because of rash	0% (0/14)	24% (5/21)	*χ^2^*(1) = 3.89, 0.049
Treatment Macrolide antibiotic Antihistamines Systemic steroids	93% (13/14) 0% (0/14) 0% (0/14)	100% (21/21) 24% (5/21) 10% (2/21)	*χ^2^*(1) = 1.54, 0.214 *χ^2^*(1) = 3.89, 0.049 *χ^2^*(1) = 1.41, 0.234
*Mp* Genotype			
P1-1/P1-2 (%) MLVA type	93%/7%	76%/24%	*χ^2^*(1) = 1.64, 0.200
MLVA-3,6,6,2 MLVA-4,5,7,2 MLVA-4,5,7,3	0% (0/12) 100% (12/12) 0% (0/12)	14% (2/14) 79% (11/14) 7% (1/14)	*χ^2^*(1) = 1.86, 0.173 *χ^2^*(1) = 2.91, 0.088 *χ^2^*(1) = 0.89, 0.345

Abbreviations: CRP, C-reactive protein; IQR, Interquartile range; MLVA, Multilocus variable-number tandem-repeat analysis; WBC, White blood cell count. Continuous variables were compared using the Mann–Whitney U-test, whereas categorical variables were compared using the Pearson chi-square test.

**Table 4:** Characteristics of patients with rare dermatological presentations of *Mycoplasma-pneumoniae*-induced cutaneous disease.

Age (yrs.)/ Gender	Dermatological Presentation	P1 Type	MLVA Type	Symptoms	Treatment	Outcome
11/M	Erythema multiforme	1	4,5,7,2	Target lesions on limbs, dehydration	azithromycin parenteral hydration	Complete recovery
6/M	Henoch–Schönlein purpura with GIT involvement	1	4,5,7,2	Abdominal pain, petechiae on lower limbs	azithromycin methylprednisolone	Complete recovery
12/M	Pityriasis rosea	2	3,6,6,2	Rash	azithromycin	Complete recovery

Abbreviations: GIT—gastrointestinal; M—male; yrs—years.

**Table 5:** Comparison of characteristics of patients admitted to the hospital because of cutaneous disease to the outpatients’ ones. Data are presented as median (IQR) or as percentage (proportion of subjects). Significant differences (*p* < 0.05) are highlighted in bold.

	Inpatients	Outpatients	Test Statistic, *p*
Subjects N	6	32	
Boys/girls (%)	50%/50%	50%/50%	*χ^2^*(1) = 0.00, 1.000
Age (years)	4.9 (IQR 3.8–7.9)	6.9 (IQR 5.0–10.0)	U(1) = 64.0, 0.200
<5 years 5–18 years	67% (4/6) 33% (2/6)	25% (8/32) 75% (24/32)	*χ^2^*(1) = 4.06, **0.044**
CRP (mg/L)	12.5 (IQR 4.0–29.0)	12.0 (IQR 4.0–39.0)	U(1) = 71.5, 0.654
WBC (× 10^9^/L)	9.8 (IQR 6.3–11.5)	9.7 (IQR 7.4–12.4)	U(1) = 72.0, 0.674
Treatment Macrolide antibiotic Antihistamines Systemic steroids	100% (6/6) 67% (4/6) 50% (3/6)	97% (31/32) 3% (1/32) 0% (0/32)	*χ^2^*(1) = 0.19, 0.661 *χ^2^*(1) = 17.85, **<0.001** *χ^2^*(1) = 17.37, **<0.001**
*Mp* Genotype			
P1-1/P1-2 (%) MLVA type	67%/33%	84%/16%	*χ^2^*(1) = 1.05, 0.305
MLVA-3,6,6,2 MLVA-4,5,7,2 MLVA-4,5,7,3	40% (2/5) 60% (3/5) 0% (0/5)	4% (1/24) 92% (22/24) 4% (1/24)	*χ^2^*(1) = 5.73, **0.017** *χ^2^*(1) = 3.49, 0.062 *χ^2^*(1) = 0.22, 0.642

Abbreviations: CRP, C-reactive protein; IQR, Interquartile range; MLVA, Multilocus variable-number tandem-repeat analysis; WBC, White blood cell count. Continuous variables were compared using the Mann–Whitney U-test, whereas categorical variables were compared using the Pearson chi-square test.

**Table 6:** Logistic regression analysis of hospital admission of *Mycoplasma-pneumoniae*-infected patients because of cutaneous disease. Significant differences (*p* < 0.05) are highlighted in bold.

	Crude OR (95%-CI)	Adjusted ^a^ OR (95%-CI)	*p* Values (Crude/Adjusted)
P1 type 1 P1 type 2	0.37 (0.05–2.60) 2.70 (0.39–18.93)	0.33 (0.04–2.48) 3.02 (0.40–22.60)	0.317/0.281 0.317/0.281
MLVA-3,6,6,2 MLVA-4,5,7,2	15.33 (1.05–224.78) 0.14 (0.01–1.36)	232.43 (1.18–45,972.69) 0.10 (0.01–1.32)	**0.046/0.043** 0.090/0.081
MLVA-4,5,7,3	0.00 (0.00)	0.00 (0.00)	1.000/1.000

^a^ Adjusted for age. Abbreviations: CI, Confidence interval; OR, Odds ratio.

## Data Availability

The data presented in this study are available on request from the corresponding author. The data are not publicly available due to privacy.

## References

[1] Atkinson T.P., Waites K.B. (2014). M*ycoplasma pneumoniae* infections in childhood. Pediatr. Infect. Dis. J..

[2] Kutty P.K., Jain S., Taylor T.H., Bramley A.M., Diaz M.H., Ampofo K., Arnold S., Williams D., Edwards K., McCullers J. (2019). *Mycoplasma pneumoniae* among children hospitalized with community-acquired pneumonia. Clin. Infect. Dis..

[3] Blasi F. (2004). Atypical pathogens and respiratory tract infections. Eur. Respir. J..

[4] Hu J., Ye Y., Chen X., Xiong L., Xie W., Liu P. (2022). Insight into the Pathogenic Mechanism of *Mycoplasma pneumoniae*. Curr. Microbiol..

[5] Schalock P.C., Dinulos J.G. (2009). *Mycoplasma pneumoniae*-induced cutaneous disease. Int. J. Dermatol..

[6] Terraneo L., Lava S.A., Camozzi P., Zgraggen L., Simonetti G.D., Bianchetti M.G., Milani G.P. (2015). Unusual eruptions associated with *Mycoplasma pneumoniae* respiratory infections: Review of the literature. Dermatology.

[7] Canavan T.N., Mathes E.F., Frieden I. (2015). *Mycoplasma pneumoniae*-induced rash and mucositis as a syndrome distinct from Stevens-Johnson syndrome and erythema multiforme: A systematic review. J. Am. Acad. Dermatol..

[8] Liakos W., Xu A., Finelt N. (2021). Clinical features of recurrent *Mycoplasma pneumoniae*-induced rash and mucositis. Pediatr. Dermatol..

[9] Chen N., Li M. (2022). Case Report and Literature Review: Clinical Characteristics of 10 Children With *Mycoplasma pneumoniae*-Induced Rash and Mucositis. Front. Pediatr..

[10] Fan X., Luo Y., Lu J., Xu J., Chen Q., Guo H., Jin P. (2021). Erythema Multiforme Major Associated With Community-Acquired Pneumonia: Lessons From a Case Report. Front. Pediatr..

[11] Dégrange S., Cazanave C., Charron A., Renaudin H., Bébéar C., Bébéar C.M. (2009). Development of multiple-locus variable-number tandem-repeat analysis for molecular typing of *Mycoplasma pneumoniae*. J. Clin. Microbiol..

[12] Dumke R., Rodriguez N. (2021). Use of different approaches for the culture-independent typing of *Mycoplasma pneumoniae* from two geographically distinct regions. J. Microbiol. Methods.

[13] Xiao J., Liu Y., Wang M., Jiang C., You X., Zhu C. (2014). Detection of *Mycoplasma pneumoniae* P1 subtype variations by denaturing gradient gel electrophoresis. Diagn Microbiol. Infect. Dis..

[14] Zhao F., Cao B., Li J., Song S., Tao X., Yin Y., He L., Zhang J. (2011). Sequence analysis of the p1 adhesin gene of *Mycoplasma pneumoniae* in clinical isolates collected in Beijing in 2008 to 2009. J. Clin. Microbiol..

[15] Dorigo-Zetsma J.W., Dankert J., Zaat S.A. (2000). Genotyping of *Mycoplasma pneumoniae* clinical isolates reveals eight P1 subtypes within two genomic groups. J. Clin. Microbiol..

[16] Sun H., Xue G., Yan C., Li S., Cao L., Yuan Y., Zhao H., Feng Y., Wang L., Fan Z. (2013). Multiple-locus variable-number tandem-repeat analysis of *Mycoplasma pneumoniae* clinical specimens and proposal for amendment of MLVA nomenclature. PLoS ONE.

[17] Brown R.J., Holden M.T., Spiller O.B., Chalker V.J. (2015). Development of a Multilocus Sequence Typing Scheme for Molecular Typing of *Mycoplasma pneumoniae*. J. Clin. Microbiol..

[18] Rodman Berlot J., Krivec U., Mrvič T., Kogoj R., Keše D. (2021). *Mycoplasma pneumoniae* P1 genotype indicates severity of lower respiratory tract infections in children. J. Clin. Microbiol..

[19] Rodman Berlot J., Mrvič T., Keše D. (2022). *Mycoplasma pneumoniae* multilocus variable-number tandem-repeat analysis genotypes are associated with inflammatory biomarker levels in children with lower respiratory tract infections. Eur. J. Clin. Microbiol. Infect. Dis..

[20] Kogoj R., Praprotnik M., Mrvič T., Korva M., Keše D. (2018). Genetic diversity and macrolide resistance of *Mycoplasma pneumoniae* isolates from two consecutive epidemics in Slovenia. Eur. J. Clin. Microbiol. Infect. Dis..

[21] Lee S.W. (2022). Methods for testing statistical differences between groups in medical research: Statistical standard and guideline of Life Cycle Committee. Life Cycle.

[22] Lee S.W. (2022). Regression analysis for continuous independent variables in medical research: Statistical standard and guideline of Life Cycle Committee. Life Cycle.

[23] Meyer Sauteur P.M., Theiler M., Buettcher M. (2020). Frequency and clinical presentation of mucocutaneous disease due to *Mycoplasma pneumoniae* infection in children with community-acquired pneumonia. JAMA Dermatol..

[24] Narita M. (2016). Classification of extrapulmonary manifestations due to *Mycoplasma pneumoniae* infection on the basis of possible pathogenesis. Front. Microbiol..

[25] Sánchez-Vargas F.M., Gómez-Duarte O.G. (2008). *Mycoplasma pneumoniae*-an emerging extra-pulmonary pathogen. Clin. Microbiol. Infect..

[26] Simmons W.L., Daubenspeck J.M., Osborne J.D., Balish M.F., Waites K.B., Dybvig K. (2013). Type 1 and type 2 strains of *Mycoplasma pneumoniae* form different biofilms. Microbiology.

[27] Lluch-Senar M., Cozzuto L., Cano J., Delgado J., Llórens-Rico V., Pereyre S., Bebear C., Serrano L. (2015). Comparative “-omics” in *Mycoplasma pneumoniae* clinical isolates reveals key virulence factors. PLoS ONE.

[28] Techasaensiri C., Tagliabue C., Cagle M., Iranpour P., Katz K., Kannan T.R., Coalson J.J., Baseman J.B., Hardy R.D. (2010). Variation in colonization, ADP-ribosylating and vacuolating cytotoxin, and pulmonary disease severity among *Mycoplasma pneumoniae* strains. Am. J. Respir. Crit. Care Med..

[29] Qu J., Yu X., Liu Y., Yin Y., Gu L., Cao B., Wang C. (2013). Specific multilocus variable-number tandem-repeat analysis genotypes of *Mycoplasma pneumoniae* are associated with diseases severity and macrolide susceptibility. PLoS ONE.

[30] Yan C., Xue G., Zhao H., Feng Y., Li S., Cui J., Ni S., Sun H. (2019). Molecular and clinical characteristics of severe *Mycoplasma pneumoniae* pneumonia in children. Pediatr. Pulmonol..

[31] Meyer Sauteur P.M., Pánisová E., Seiler M., Theiler M., Berger C., Dumke R. (2021). *Mycoplasma pneumoniae* genotypes and clinical outcome in children. J. Clin. Microbiol..

[32] Zhou Y., Zhang Y., Sheng Y., Zhang L., Shen Z., Chen Z. (2014). More complications occur in macrolide-resistant than in macrolide-sensitive *Mycoplasma pneumoniae* pneumonia. Antimicrob. Agents Chemother..

